# Development and evaluation of an effective solubility prediction model for pharmaceuticals in organic solvents using machine learning based on eXtreme Gradient Boosting

**DOI:** 10.1038/s41598-026-53038-w

**Published:** 2026-05-28

**Authors:** Masood Valavi, Mehdi Assareh, Ali Khoshsima, Michael Svärd

**Affiliations:** 1 Thermodynamics Research Laboratory, Iran University of Science and Technology, Tehran, Iran; 2 Faculty of Petroleum and Chemical Engineering, Hakim Sabzevari University, Sabzevar, Iran; 3 Department of Chemical Engineering, KTH Royal Institute of Technology, Stockholm, Sweden

**Keywords:** Solubility prediction, Pharmaceuticals, Temperature, Machine learning, Chemistry, Drug discovery, Mathematics and computing

## Abstract

In this work, we have examined the predictive capability of a machine leaning model based on the XGBoost framework as regards the solubility of active pharmaceutical ingredient-like molecules in organic solvents over a wide range of temperatures. A total of 30 binary mixtures has been investigated. The dataset was divided in two sets, with one set for training, testing and validation including solubility data for four solute compounds (butyl paraben, fenofibrate, risperidone, fenoxycarb) consisting of a total of 224 data points, and the second set used for prediction consisting of the solubility data for butamben, with 50 data points in total. The calculated root mean square errors (RMSLE) for the calculated solubility (train, test, validation) were 0.05, 0.09, 0.13 and 0.15, respectively, while the average RMSLE for the predicted solubility of butamben was 0.41. A total of 10 descriptors were considered in this work, comprising parameters for solute (heat of fusion, melting temperature, heat capacity and Hansen solubility parameter), two descriptors representing the solvent (dielectric constant and boiling temperature) as well as temperature, and drug and solvent names. The temperature-dependence of solubility has been captured accurately by setting a constraint on the XGBoost algorithm. A comparison between the performance of the machine learning model proposed and evaluated in this work, and the semi-predictive Flory-Huggins and the temperature-dependent NRTL-SAC models on the other hand, shows that for all the studied compounds, the machine learning model can deliver significantly improved capability to model as well as predict solubility.

**Supplementary Information:**

The online version contains supplementary material available at 10.1038/s41598-026-53038-w.

## Introduction

Solvent selection is an important step in crystallization of active pharmaceutical ingredients (APIs), as the solvent apart from the yield can significantly influence the properties of the crystallized product. In this regard, accurate data on the solubility of APIs in organic and inorganic solvents is a key part of the development of a crystallization process, essential for proper solvent selection^1^. Considering that experimental determination of solubility from scratch can be tedious and expensive, alternative approaches such as thermodynamic models are in high demand.

There are two kinds of models for predictive calculation of solubility: one is based on classical thermodynamic models while the other is based on machine learning algorithms. The first can be further divided into three types:

(i) Semi-empirical models; e.g. NRTL, UNIQUAC, and the Wilson model. These depend on fitting to experimental data for drug-solvent mixtures using adjustable parameters.
(ii) Semi-predictive models; e.g. UNIFAC, NRTL-SAC (original and temperature-dependent), Flory-Huggins, PC-SAFT, and UNIQUAC-SAC. Semi-predictive approaches such as NRTL-SAC^2,3^ and the Flory-Huggins^4^ model use a portion of data to calibrate the model.
(iii) Completely predictive models; e.g. COSMO.

The correlative semi-empirical models can show good performance for specific systems - for example, Mirmehrabi et al.^5^ fitted the parameters of the UNIQUAC model to model the solubility of two polymorphs of ranitidine hydrochloride and obtained accuracies of 0.89% and 3.53%, respectively. On the other hand, completely predictive models are often limited by missing data and high computational costs^3,4^. For instance, COSMO-RS^6^ and COSMO-SAC^7^ have shown moderate capability with RMSLEs of 1.6 to 3.2. Overall, semi-predictive models can offer a good tradeoff between high accuracy and wide generalizability without excessive cost. Although the UNIFAC^8^ model does not include many of the interaction parameters of several APIs, it typically shows good prediction ability for many API-solvent mixtures. Valavi et al.^3,4^ used NRTL-SAC and Flory-Huggins models and obtained average RMSLEs of 1.09 and 0.91 for a set of compounds including butyl paraben, fenoxycarb, fenofibrate and risperidone, respectively. Ruther and Sadowski^9^ calibrated PC-SAFT parameters using data for solubility of paracetamol, ibuprofen, sulfadiazine, *p*-hydroxyphenylacetic acid, and *p*-aminophenylacetic acid from a single solvent resulting in reasonable prediction for other solvents.

Models based on machine learning (ML) have recently been used to predict the solubility of pharmaceutical compounds. Ghanavati et al.^10^ utilized a random forest-based feature selection on tabular features, followed by mapping with eXtreme Gradient Boosting (XGBoost). The proposed ML-based models, trained on 80% of each dataset and evaluated on the remaining 20%, showed superior performance, particularly with XGBoost utilizing the extracted and selected tabular features. This yielded average test data mean absolute error (MAE), root mean squared error (RMSE), and R-squared (*R*^2^) values of 0.458, 0.613, and 0.918, respectively. They also used an ensemble of the three models and showed improvement in error metrics across all datasets, consistently outperforming each individual model used separately. This ensemble model was also tested in the Solubility Challenge 2019^11^ achieving an RMSE of 0.865 and outperforming 37 models with an average RMSE of 1.62. Boobier et al.^12^ reported a successful approach to solubility prediction in organic solvents and water using a combination of machine learning (Artificial Neural Network (ANN), Support Vector Machine (SVM), Random Forest (RF), Extra Trees (ET), Bagging and Gaussian Process (GP)) and computational chemistry. The models employed in their work gave accurate predictions with accuracies close to the expected level of noise in the training data (log *S* ± 0.7). Finally, they reproduced physicochemical relationships between solubility and molecular properties in different solvents, which led to rational approaches to improve the accuracy of each model. In the study of Ye and Ouyang^13^ molecular fingerprints were selected to characterize structural features. Light Gradient Boosting Machine (LightGBM) was compared with deep learning and traditional machine learning (Partial Least Squares (PLS), ridge regression, RF, SVM) to develop models for predicting solubility in organic solvents at different temperatures. It is reported that compared to other models, LightGBM exhibited significantly better overall generalization (log *S* ± 0.20). For unseen solutes, their proposed model gave a prediction accuracy (log *S* ± 0.59) close to the expected noise level of experimental solubility data. Yousefi and Movagharnejad^14^ used three different artificial neural networks including multilayer perceptron, radial basis function (RBF), and SVM, to predict the solubility of different pharmaceutical compounds in water and various solvents. Molecular weight, melting point, temperature, and the number of each functional group in the pharmaceutical compound and organic solvents were selected as the input variables of these three different neural network models. Ge and Ji^15^ proposed a novel strategy that combined molecular thermodynamics and machine learning to accurately predict the solubility of drugs in various solvents. The strategy was based on 16 molecular descriptors representing drug–drug interactions and drug–solvent interactions, including physical parameters, pure perturbed-chain statistical associating fluid theory (PC-SAFT) parameters of drugs and solvents, and mixing rules.

Kyhoiesh et al.^16^ used a ML technique to model solubility in compounds related to ibuprofen. Linear and RF regression achieved remarkable predictive power, with *R*^2^ values ranging from 0.86 to 0.92 and RMSE values between 0.002 and 0.34. They reported that, for compounds with solubilities exceeding 80 g L^− 1^, solubility mapping revealed a significant correlation between number of hydroxyl groups and enhanced solubility. Their study illustrates the potential for ML-driven design to streamline pharmaceutical development, predicting aqueous solubility prior to manufacturing and conserving valuable resources. Mahdi et al.^17^ employed ML-based methods to predict salicylic acid solubility in various solvents as a function of pressure and temperature. Using a dataset consisting of 217 data points and 15 input features, the analysis was performed using variables including pressure, temperature, and 13 different solvents as integral aspects. They used convolutional neural networks (CNNs), polynomial regression (PR), and kernel ridge regression (KRR), to predict the solubility of salicylic acid. The hyperband method was utilized for hyper-parameter optimization, ensuring optimal performance for each model by dynamically allocating computational resources. Alqarni and Alqarni^18^ investigated the use of ML models to predict the solubility of rivaroxaban in binary solvents based on temperature, mass fraction and solvent type. Using a dataset with over 250 data points and including solvents encoded with one-hot encoding, four models were compared: GB, LGB, ET and RF. A summary of important works on predictive solubility modelling of pharmaceuticals in organic solvents is given in Table 1.

**Table 1:** Summary of studies on prediction of drug solubility in organic solvents.

Authors, reference	Title	Key Findings
Mirmehrabi, Rohani, Perry^19^	Thermodynamic Modeling of Activity Coefficient and Prediction of Solubility: Part 2. Semipredictive or Semiempirical Models	Proposed a modified semi-predictive model with 2 adjustable parameters (a₁, a₂). Predictions with NRTL using one experimental point yielded large errors, while the modified model and UNIQUAC were superior. Developed a method to predict solubility of all polymorphs using data from just one polymorph. UNIQUAC was marginally superior, but the new model is simpler and does not require molecular parameters.
Tung, Tabora, Variankaval, Bakken, Chen^20^	Prediction of Pharmaceutical Solubility Via NRTL-SAC and COSMO-SAC	Compared NRTL-SAC and COSMO-SAC for solubility estimation. NRTL-SAC showed better performance (avg. log₁₀ square error 0.105 vs. 0.318). Successfully predicted solubility in mixed solvents using parameters from pure solvents. COSMO-SAC required re-optimization for larger molecules.
Ruether, Sadowski^9^	Modeling the Solubility of Pharmaceuticals in Pure Solvents and Solvent Mixtures for Drug Process Design	Applied PC-SAFT using limited experimental data. Required only two solubility points per solvent class. Introduced temperature-dependent binary interaction parameters. Successfully predicted solubility in solvent mixtures using only pure-solvent data.
Abraham, Acree, et al.^21^	Prediction of Solubility of Drugs and Other Compounds in Organic Solvents	Used Abraham solvation equations (LFER). Solute descriptors derived from a few solvents allow prediction in ~ 85 solvents. Particularly effective for highly hydrophobic compounds.
Nouar, Benmessaoud, et al.^22^	Solubility Prediction of Active Pharmaceutical Compounds with the UNIFAC Model	Developed a new-based UNIFAC combining Pharma-UNIFAC and KT-UNIFAC. Estimated over 100 interaction parameters. Reduced average relative deviation from ~ 67% to ~ 9%.
Schroeter, Schwaighofer, et al.^23^	Estimating the Domain of Applicability for Machine Learning QSAR Models: A study of aqueous solubility of drug discovery models	Compared GP, SVM, RF, and Ridge Regression. Error bars defined the domain of applicability. Rejecting out-of-domain compounds improved external prediction accuracy. GP produced the most reliable uncertainty estimates.
Faller, Ertl^24^	Computational Approaches to Determine Drug Solubility	Identified logP as key but insufficient alone. Tautomerism caused major prediction errors. A 3-parameter model (logP, PSA, relative volume) achieved r² = 0.726. Predictive power decreased in modern drug-like solubility ranges.
Nordstrom, Rasmuson^25^	Prediction of Solubility Curves and Melting Properties of organic and pharmaceutical compounds	Demonstrated prediction of melting temperature from solubility data. With one solubility point and melting data, full solubility curves can be predicted. Heat capacity effects are significant.
Mota, Carneiro, et al.^26^	Temperature and solvent effects in the solubility of some pharmaceutical compounds: Measurements and modeling	Measured solubility of four drugs in six solvents. NRTL-SAC achieved 68% AAD for organics and 38% for water. Suitable for single and mixed solvent systems.
Spyriouni, Krokidis, Economou^27^	Thermodynamics of pharmaceuticals: Prediction of solubility in pure and mixed solvents with PC-SAFT	Parameterized PC-SAFT using three solvents. Used scaled aqueous solubility. Achieved reasonable predictions without extra binary parameters except for water.
Mota, Queimada, et al.^28^	Solubility of drug-like molecules in pure organic solvents with the CPA EoS	First application of CPA EOS to drug solubility. Used one binary interaction parameter. Explicitly modeled association effects.
Bouillot, Teychené, Biscans^29^	An evaluation of thermodynamic models for the prediction of drug and drug-like molecule solubility in organic solvents	Compared UNIFAC, COSMO-SAC, and NRTL-SAC. UNIFAC gave best order-of-magnitude estimates. COSMO-SAC overestimated solubility when hydrogen bonding was important.
Mota, Queimada, Andreatta, et al.^30^	Calculation of drug-like molecules solubility using predictive activity coefficient models	Extended A-UNIFAC with new interaction parameters. NRTL-SAC performed best at 298 K. Reference Solvent Approach significantly improved predictions.
Matsuda, Mori, et al.^31^	Determination and prediction of solubilities of active pharmaceutical ingredients in selected organic solvents	Measured solubility of multiple polymorphs. Pharma-Modified UNIFAC outperformed modified UNIFAC. Identified stable and metastable polymorphs via solubility data.
Hahnenkamp, Graubner, Gmehling^32^	Measurement and prediction of solubilities of active pharmaceutical ingredients	Compared UNIFAC and COSMO-RS. UNIFAC underestimated and COSMO-RS overestimated solubility. Modified UNIFAC best at solvent ranking.
Svärd, Rasmuson^33^	(Solid+ liquid) solubility of organic compounds in organic solvents–Correlation and extrapolation	Developed a semi-empirical model specifically including ΔC_p_ effects, and accounting for temperature-dependence of van’t Hoff enthalpy. Proposed a robust 3-parameter extrapolation model.
Valavi, Ukrainczyk, Dehghani^4^	Prediction of solubility of active pharmaceutical ingredients by semi-predictive Flory Huggins/Hansen model	Used Hansen parameters within Flory–Huggins theory. Molecular dynamics validated fitted parameters. Accuracy decreased with increasing molecular weight.
Chen, Crafts^2^	Correlation and prediction of drug molecule solubility in mixed solvent systems with the nonrandom two-liquid segment activity coefficient (NRTL − SAC) model	Introduced NRTL-SAC with conceptual segments. Requires 4–8 experimental points. Suitable for early solvent screening.
Valavi, Svärd, Rasmuson^3^	Prediction of the solubility of medium-sized pharmaceutical compounds using a temperature - dependent NRTL - SAC model	Extended NRTL-SAC with temperature-dependent interactions. Improved solubility predictions compared to original NRTL-SAC and Pharma-UNIFAC.
Ran, Yalkowsky^34^	Prediction of drug solubility by the general solubility equation (GSE)	Validated revised GSE. Outperformed complex models. Melting point term was essential.
Gracin, Brinck, Rasmuson^35^	Prediction of solubility of solid organic compounds in solvents by UNIFAC	UNIFAC is often inaccurate. Quantum calculations showed functional group non-independence. Showed that neglect of ΔC_p_ contributed to errors.
Sheikholeslamzadeh, Rohani^36^	Solubility prediction of pharmaceutical and chemical compounds in pure and mixed solvents using predictive models	NRTL-SAC outperformed UNIFAC. Accurate prediction of solubility maxima in mixed solvents.
Bouillot, Teychené, Biscans^37^	Discussion and improvement of the refined cosmo-sac parameters for solubility predictions: Part 2	Re-optimized hydrogen bonding parameters. Improved RMSE. Showed solute interaction sites are partially hidden.
Modarresi, Conte, et al.^38^	Model -based calculation of solid solubility for solvent selection: A review	Reviewed solvent-selection models. Introduced UNIFAC-CI and new GC-CI models. Built a large SLE database.
Boobier, Hose, Blacker, Nguyen^12^	Machine learning with physicochemical relationships : solubility prediction in organic solvents and water	CSPR approach using few descriptors. ML outperformed commercial tools. Predictions close to experimental noise.
Cysewski, Jeliński, et al.^39^	Solubility of Sulfanilamide and Sulfacetamide in neat solvents: Measurements and interpretation using theoretical predictive models, first principle approach	Evaluated eight models. Buchowski–Ksiazczak model performed best. Hybrid ML models improved COSMO-RS predictions.
Ge, Ji^15^	Novel computational approach by combining machine learning with molecular thermodynamics for predicting drug solubility in solvents	Combined PC-SAFT parameters with ML. Binary interaction parameter was most important descriptor.
Zarei Mahmoudabadi, Pazuki^40^	A predictive PC-SAFT EOS based on COSMO for pharmaceutical compounds	Fully predictive PC-SAFT model derived from COSMO data. Improved accuracy over COSMO-SAC.

Despite the extensive body of work done on pharmaceutical solubility modelling, there are still some important gaps remaining. Many existing approaches rely either on large experimental datasets, extensive compound-specific parameter fitting, or descriptors that are difficult to obtain in early-stage development. Moreover, the predictive performance is often assessed using metrics that are not easily interpretable in terms of experimental uncertainty, and external validation on fully unseen APIs remains limited. In particular, there is a lack of physically interpretable and data-efficient modeling frameworks that can reliably predict temperature-dependent solubility while maintaining acceptable accuracy for solvent screening applications. Addressing these limitations motivates the present study, which aims to develop and validate a predictive model with clear thermodynamic relevance and robust extrapolative capability. Specifically, the aim of the present work is to assess the potential of a machine learning model based on the XGBoost algorithm to predict the solubility of a typical API, butamben, in organic solvents by training and validating the model on a dataset of solubilities of four other organic solutes in organic solvents across a range of temperatures.

## Methods

The solute-solvent systems included in this work are listed in Table 2. In total, 30 binary solute-solvent systems have been examined in this work to assess the model performance. With temperatures in the range 268 K to 323 K, a total of 224 data points have been used.

**Table 2:** Model compounds and solvents included in this work.

Solute	Solvents
butyl paraben	methanol, ethyl acetate, ethanol, 1-propanol, acetonitrile, acetone
fenoxycarb	methanol, toluene, ethanol, 2-propanol, ethyl acetate
fenofibrate	methanol, ethyl acetate, ethanol, 1-propanol, 2-propanol, acetonitrile
risperidone	methanol, toluene, ethanol, 1-propanol, 2-propanol, 1-butanol, acetone, ethyl acetate
butamben	methanol, 1-propanol, 2-propanol, 1-butanol, toluene

### Preprocessing and feature engineering

The first step when designing and using ML-based methods is data preprocessing. Our survey shows that there are no missing values in the input parameters to the model meaning that all input variables are well reported in the literature of selected studies. Categorical inputs such as drug name and solvent name were converted to numerical values using one-hot encoding. Numerical features such as temperature were considered as continuous features. In addition to categorical features and temperature, the following physicochemical features were considered as model inputs: solute heat of fusion, solute melting point, heat capacity of solute (two parameters), Hansen solubility parameter of solute, solvent dielectric constant and solvent boiling point. For the heat capacity of the solute, this was covered through the temperature-dependent difference between the heat capacities of solid and liquid phase. We specifically chose to consider the temperature dependence of the heat capacity since our previous works show this to be important for predictive modelling of solubility. A two-parameter linear regression equation was used, Eq. (1):

1 $$\Delta {\text{Cp = q + r}(T - T_m)}$$

The parameters used for the different solute molecules are listed in Table 3.

**Table 3:** Model compound-dependent features used in this work.

Solute	Tₘ (K)	ΔH_fus_(Tₘ) (kJ mol⁻¹)	q (J K⁻¹ mol⁻¹)	*r* (J K⁻² mol⁻¹)	Hansen solubility parameter	References
butyl paraben	340.5	26	77.2	0.490	21.5	^4,41^
fenoxycarb	326.3	26.98	106.5	0.0424	26.08	^4,42^
fenofibrate	352.05	33.5	124.3	0.5192	19.72	^4,43^
risperidone	442.4	43.9	158.1	0.5214	21.87	^4,44^
butamben	330	74.12	30	0.86	20.63	^4,45^

In this work the solvent-specific parameters included are the dielectric constant and boiling temperature. The reason for including the dielectric constant is to capture the polarity of the solvent. Conversely, the boiling temperature of solvent in a way represents the strength of intermolecular interactions, notably the hydrogen bond network, between molecules in the pure solvent condensed phases. These solvent descriptors were selected to reflect the primary intermolecular van der Waals’ interactions controlling pharmaceutical solubility, namely hydrogen bonding and other electrostatic forces, together with Debye and London/dispersion forces. Only descriptors with clear physical meaning and broad availability were retained to ensure model interpretability and applicability in solvent screening scenarios. Additional solvent features were evaluated but excluded due either to strong correlation with the selected descriptors or negligible improvement in predictive accuracy, in order to avoid overfitting and to maintain model robustness. Dielectric constants and boiling temperatures of the included solvents are listed in Table 4. As can been seen from Table 4, a wide range of solvents of different polarity has been included in this work, ranging from very polar solvents such as methanol and acetonitrile to strongly non-polar solvents such as toluene. All solvent-specific data used in this study is taken from reference^46^.

**Table 4:** Dielectric constants and boiling points of the solvents included in the study.

Solvent	Dielectric constant	Boiling point (K)	Reference
methanol	35.2	337.8	^46^
ethanol	24.8	351.2	^46^
1-propanol	20.5	370.4	^46^
2-propanol	17.4	355.6	^46^
1-butanol	15.7	390.7	^46^
acetone	19.5	329.4	^46^
ethyl acetate	5.4	350.8	^46^
toluene	2.2	383.2	^46^
acetonitrile	34.1	355.3	^46^

The entire dataset was then divided into two parts. The first subset includes data for binary mixtures of four compounds (butyl paraben, fenofibrate, risperidone and fenoxycarb) with organic solvents at different temperatures. The resulting subset consists of 224 data points in total. This dataset was randomly divided into training and testing subsets, with 80 per cent of the data used for training and the remainder for testing. The partitioning was performed in a way to ensure consistent distribution of solvents and temperatures across training and testing sets. For the evaluation strategy a grouped splitting approach was implemented, specifically [leave-one-solute-out/leave-one-solvent-out/grouped solute–solvent pair split]. The second subset included solubility data for the solute butamben in five different organic solvents at different temperatures (50 data points in total). This dataset was used as an external test case, for which the solubility data is completely excluded from the data used to train the model. This was done to evaluate to what extent the trained model can be used to predict the solubility of an entirely new compound.

It is worth mentioning that standardization was used for both training and testing in order to normalize the datasets to reduce sensitivity to the scale of input features.

### Model selection

For this work, an ensemble tree-based method was selected; eXtreme Gradient Boosting (XGBoost). XGBoost has been shown to be a very powerful model for its efficiency and accuracy for solubility prediction^10^. It has a high capability and flexibility for regularization to manage complex data making it suitable for solubility prediction that involves heterogeneous feature types.

The solubility of API-type molecules in organic solvents generally increases monotonically with temperature. To capture this fact, a positive monotonic constraint was applied specifically to the temperature feature column, a feature allowed for in the XGBoost method. This constraint ensures that the predicted solubility increases with increasing temperature.

### Hyperparameter optimization

For the purpose of optimizing model performance, a grid search approach was used to tune critical parameters. Five-fold cross-validation on the training data was incorporated into the grid search. As a result of this, the best model configuration was selected based on highest average validation score. The obtained optimized parameters are listed in Table 5. Finally, the best optimized model was used to predict the solubility of butamben in five organic solvents over a range of temperatures.

**Table 5:** Optimized XGBoost model parameters.

Parameter	Value
*n_estimators*	300
*max_depth*	4
*learning_rate*	0.05
*subsample*	0.7
*colsample_bytree*	0.7
*random_state*	42
*objective*	reg: squarederror
*booster*	gbtree
*gamma*	0
*reg_alpha*	0
*reg_lambda*	1
*min_child_weight*	1
*tree_method*	auto

### Model evaluation

In this work, in order to accurately account for the large variety between solubility values of different solutes in different solvents (spanning several orders of magnitude), the logarithmic value of solubility was used as target value for model training. Furthermore as in our previous work^3,4^, the root mean squared logarithmic error (RMSLE; Eq. (2)) was used to assess and grade model performance.

2 $$\mathrm{RMSLE}=\frac{\sqrt{\sum\:{({\mathrm{ln}x}^{\mathrm{exp}}-{\mathrm{ln}x}^{\mathrm{calc}})}^{2}}}{\mathrm{N}}$$

This choice has the benefit of facilitating comparison between this and previous works. In addition to RMSLE, the mean absolute percentage deviation (MAPD) was calculated to provide a metric directly comparable with the relative standard deviation of experimental solubility measurements. While RMSLE was retained as the primary metric due to the wide solubility range across systems, MAPD offers a more intuitive measure of predictive accuracy from an experimental perspective. It is worth mentioning that, except for butamben, the same solute-solvent binary mixtures have been evaluated in the works cited for comparison. RMSLEs for each drug-solvent binary mixture for both training and testing sets were computed, and the model performance was then evaluated using the same statistics for prediction of the solubility of butamben. The latter step is crucial for evaluation of the capability of a model for generalization and prediction of the solubility of new compounds.

## Results

The RMSLE as defined in Eq. (2) was used consistently for assessing model performance. The average RMSLE values for all binary solute-solvent mixtures in the whole dataset (train, validation, test) obtained with the XGBoost model following parameter optimization are listed in Table 6. The table also lists RMSLEs obtained in previous studies for comparison^3,4^.

**Fig. 1:**
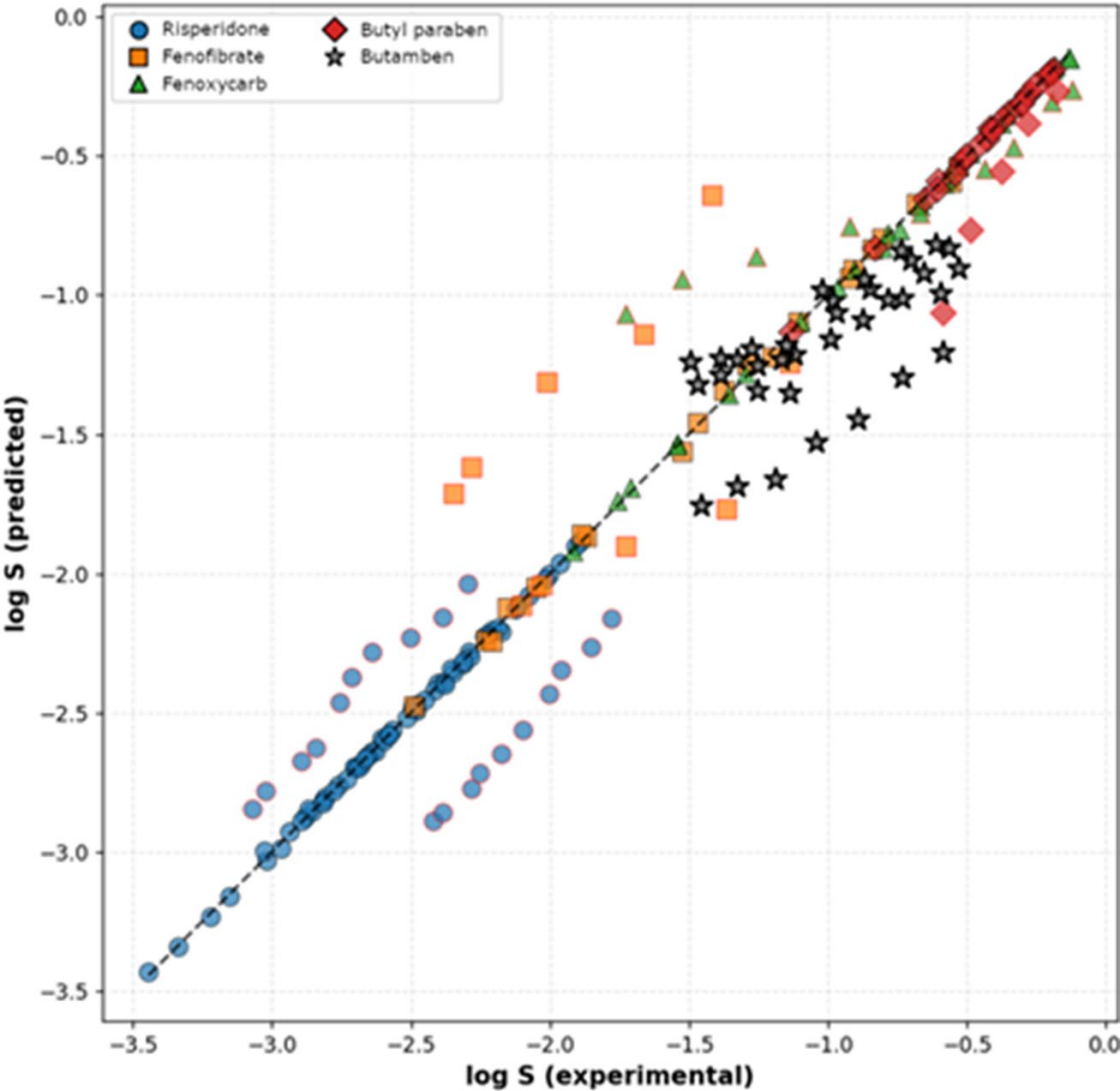
Calculated vs. experimental (logarithm of mole fraction) solubility in organic solvents showing the performance of the optimized XGBoost-based machine learning model for butyl paraben, fenoxycarb, fenofibrate and risperidone (training and testing sets).

Figure 1 shows the relationship between experimental values and predicted values for all four solutes included in the train and test datasets, obtained with the machine learning model. It is clear from Table 6 as well as Fig. 1 that the optimized XGBoost-based model is capable of reproducing solubilities (as logarithms of the mole fraction) with excellent accuracy, with *R*^2^ values for the training set equal to 0.98 and for the test set equal to 0.86.

Finally, for the verification dataset encompassing the solubility of a new solute, butamben, in five solvents, the average RMSLE obtained using the optimized model across all solvents was 0.41, with RMSLEs of the solubility in methanol, 1-propanol, 2-propanol, 1-butanol and toluene of 0.22, 0.25, 0.31, 0.60 and 0.65, respectively.

**Table 6:** RMSLE for the predicted solubility of solute-solvent mixtures obtained using the XGBoost model, with data obtained with the FH1, FH2 and NRTL-SAC models in previous work^3,4^ listed for comparison.

Solute	Solvent	RMSLE				MAPD (%)	
		FH1^4^	FH2^4^	NRTL-SAC^3^	XGBoost	XGboost	*T*-range (K)
butyl paraben	methanol	0.05	0.04	0.02	0.03	3.23	283–323
	ethyl acetate	0.15	0.05	0.05	0.032	2.14	283–323
	ethanol	0.66	0.19	0.2	0.048	4.43	283–323
	1-propanol	0.12	0.35	0.09	0.048	2.25	283–323
	acetonitrile	0.2	0.22	0.5	0.038	5.56	283–323
	acetone	0.19	0.14	0.03	0.120	2.11	283–323
	Average	0.23	0.17	0.14	0.05	3.14	
fenoxycarb	methanol	0.76	0.62	0.02	0.17	53.32	278–318
	toluene	0.3	0.05	0.17	0.13	6.45	278–318
	ethanol	0.47	0.56	0.45	0.04	2.98	278–318
	2-propanol	0.2	1.08	1.61	0.01	14.11	278–318
	ethyl acetate	0.15	0.52	0.02	0.13	5.61	278–318
	Average	0.38	0.57	0.45	0.09	18.35	
fenofibrate	methanol	0.52	0.86	0.04	0.04	4.12	278–318
	ethyl acetate	0.81	0.17	0.12	0.49	65.46	278–318
	ethanol	0.7	0.8	0.59	0.02	5.84	278–318
	1-propanol	0.55	0.8	1.43	0.01	4.32	278–318
	2-propanol	0.31	1.81	2.97	0.22	23.41	278–318
	acetone	1.07	1.21	1.39	0.01	6.90	278–318
	Average	0.66	0.94	1.09	0.13	17.52	
risperidone	methanol	2.01	0.45	0.08	0.05	2.14	278–323
	toluene	0.72	0.02	0.14	0.38	139.25	278–323
	ethanol	0.29	0.31	1.22	0.05	2.15	278–323
	1-propanol	0.04	1.06	2.39	0.020	1.92	278–323
	2-propanol	1.12	2.58	1.87	0.01	2.53	278–323
	1-butanol	0.13	2.55	1.47	0.03	1.14	278–323
	acetone	1.22	4.27	4.23	0.34	1.78	278–323
	Average	0.6	1.6	1.4	0.15	38.41	

## Discussion

### Solute-specific model performance

Among all examined solute compounds, the evaluated model exhibited the best performance for butyl paraben, where the mole fraction solubility varies approximately between 0.05 and 0.7. For this compound, the average RMSLE obtained was 0.05 (train + test). Low RMSLEs were obtained for all six evaluated solvents (in the range 0.03 to 0.12), showing that the model is consistent across different solvents. It is worth mentioning that both the Flory Huggins and the NRTL-SAC models also showed the best performance for this compound, although both resulting in significantly larger errors, as shown in Table 6. For butyl paraben the largest errors are observed for the cases with the lowest solubility, in which cases the machine learning model tends to overestimate the solubility. But overall, calculated values are in good accordance with experimental values.

The XGBoost-based model is also able to calculate the solubility of fenoxycarb in the evaluated solvents with an overall high accuracy, with an average RMSLE equal to 0.09. In particular, very low errors were obtained for the solubility in 1-propanol (0.01; see Table 6). The RMSLEs obtained for other binary fenoxycarb systems vary between 0.04 and 0.17.

For the compound fenofibrate, the model also performs well, with an average RMSLE of 0.13. Again, this shows the capability of XGBoost as a powerful model to capture even the very low solubility values reported for this compound. In Table 6, it can be seen that the lowest error was observed in acetone (0.01) while the worst result was obtained for ethyl acetate (0.49). For ethyl acetate, two of the compared models perform better, although overall across all solvents the XGBoost-based model is capable of calculating the solubility of fenofibrate with better accuracy.

Finally, the average RMSLE obtained for the XGBoost model when modelling the solubility of risperidone is 0.15. Overall, as Table 6 shows, the superior performance of the machine learning model in comparison to the Flory Huggins and NRTL-SAC models is particularly clear for this compound. The largest error was observed for toluene with an RMSLE of 0.38. For acetone, although the RMSLE is high (0.34), it is approximately an order of magnitude lower than values obtained for the compared models. It should be emphasized that this compound exhibits mole fraction solubilities 1–2 orders of magnitude lower than the other compounds. This results from a combination of low solute activity and strongly non-ideal interactions with the evaluated solvents^44^ and is likely a strong reason why the solubility has been difficult to accurately model with the simpler Flory Huggins and NRTL-SAC models.

One important observation is that the errors are closely tied to the combination of solute and solvent. For some combinations, the error can be quite high, while values for the same compound in other solvents, as well as values for other solutes in that solvent, can be much lower. This should not be surprising, each combination being a chemically unique system, and shows that for solubility calculations it is the interplay of both solute and solvent properties which is most important.

### Generalizing the model: Predicting the solubility of a new compound

**Fig. 2:**
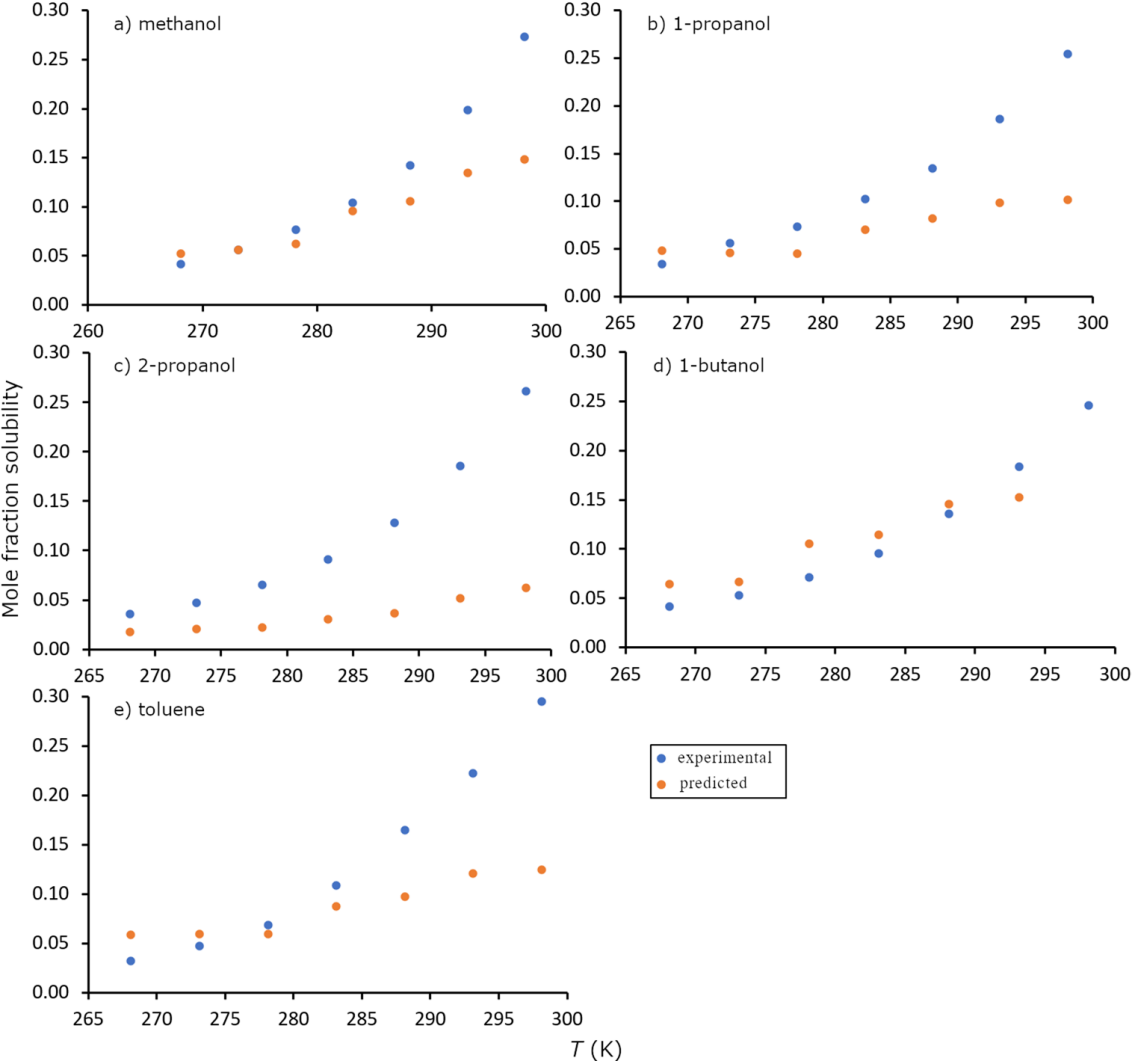
Predicted vs. experimental (mole fraction) solubility curves of butamben in (**a**) methanol, (**b**) 1-propanol, (**c**) 2-propanol, (**d**) 1-butanol and (**e**) toluene.

The solubility of butamben in five organic solvents was used as a case to evaluate the capability of the XGBoost model to predict the solubility of a compound outside the test set. The average RMSLE obtained in methanol, 1-propanol, 2-propanol, 1-butanol and toluene over the temperature range 268 K to 323 K is 0.41, obtained for prediction of solute-solvent binary mixtures. Figure 2a-e show the predicted solubility compared with the experimentally determined values for butamben in the five organic solvents. Although the obtained RMSLE is higher than for the training set solutes, the corresponding MAPD of 65% remains within a range comparable to typical experimental uncertainty reported for solubility measurements, indicating acceptable predictive capability for solvent screening purposes.

**Fig. 3:**
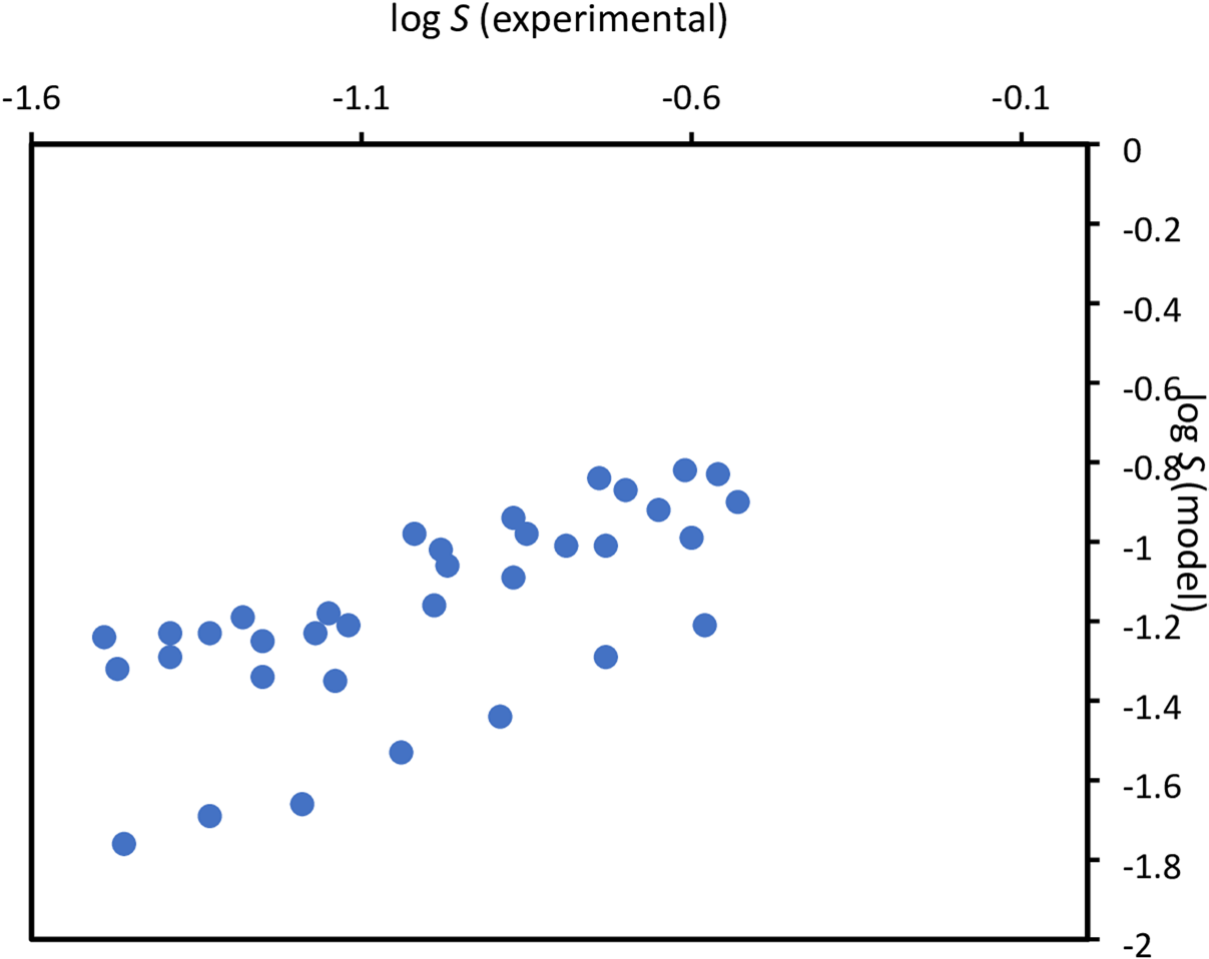
Predicted vs. experimental (logarithm of mole fraction) solubility in organic solvents showing the predictive performance of the optimized XGBoost-based model for butamben.

Figure 3. shows a comparison between the experimental and predicted solubility values of butamben across all solvents. 

### Comparison with previous works

The proposed model based on machine learning presents comparatively superior accuracy when compared to semi-predictive models such as the traditional Flory-Huggins and NRTL-SAC models, as shown in Table 6. The average RMSLE over four compounds for FH1, FH2 and NRTL-SAC is equal to 0.44, 0.75 and 0.74, respectively, while for the proposed XGBoost-based model the corresponding average RMSLE is 0.08. It should be mentioned here, however, that – although the same experimental data is used – the results obtained using the XGBoost-based model and the Flory Huggins and NRTL-SAC models, respectively, were obtained under different training–testing splits, and thus do not constitute a strictly controlled head-to-head comparison.

**Fig. 4:**
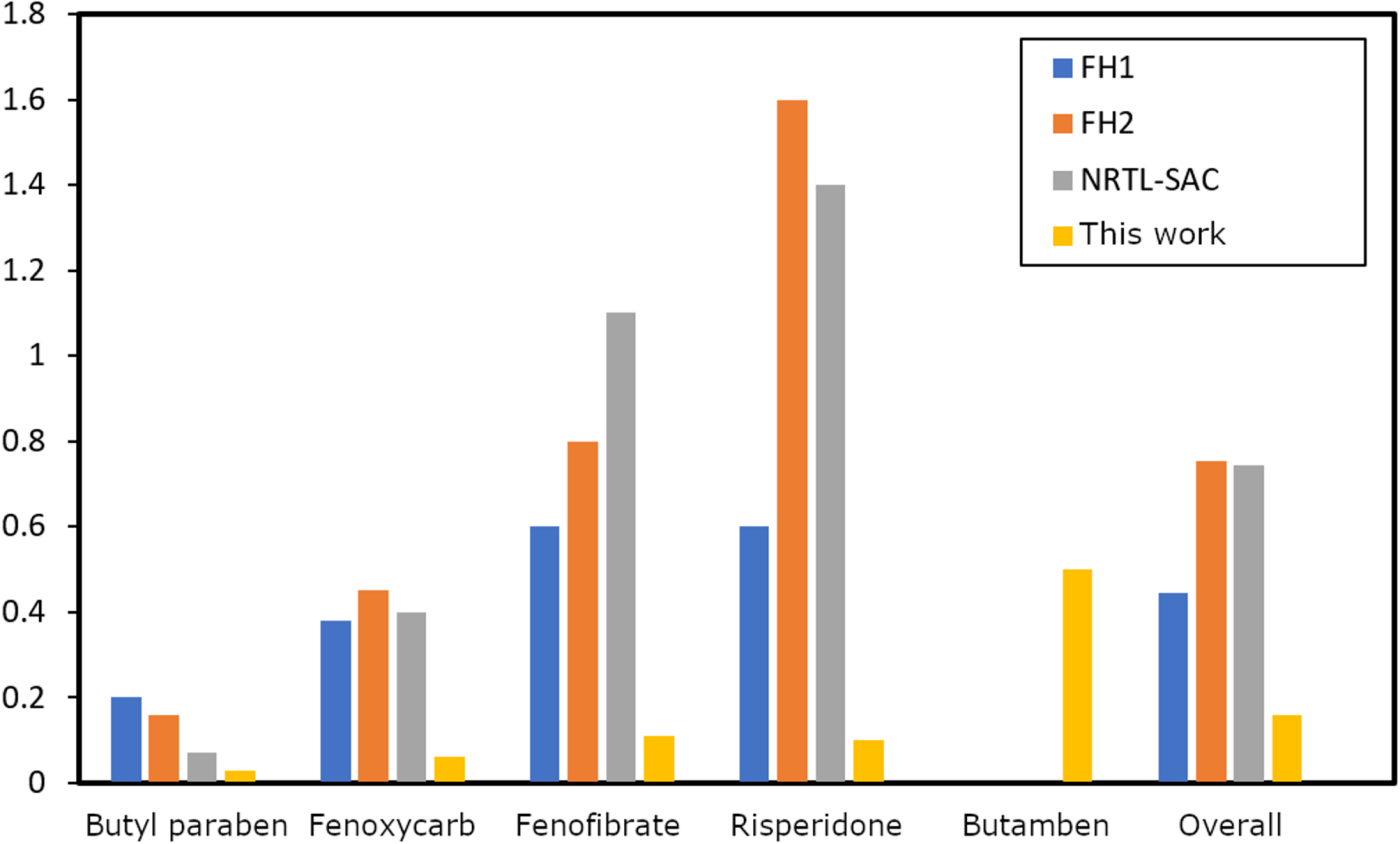
Average RMSLEs for calculated solubilities of four compounds obtained with four different models.

As Fig. 4 shows, for all solutes the XGBoost model provides superior performance in comparison to the other classical models. In particular, this can be observed in case of the compound risperidone where the error is approximately one order of magnitude smaller. Similar small errors are obtained for all four solute molecules using the new model. Finally, it can be observed that for the pure prediction for a new solute (butamben), although – as can be observed e.g. by comparing the scatter plots in Figs. 1 and 3 – the predicted accuracy is still far from perfect, with larger RMSLE than for the training + test data, the error *for prediction* is now similar in magnitude to the results obtained for training + test sets using the classical models.

In similarity to other reported works using machine learning models, the model employed in this work is capable of modelling solubility data with good accuracy. For example, Fowles et al^47^. employed the Extra Trees model for calculation of the solubility of succinic acid, desloratadine and coronene. The best result obtained was for succinic acid with a MAPD of 20%, which may be compared to 18% obtained in the present study across all systems and temperatures.

### Benefits of the machine learning model and further improvement

Machine learning models, such as the presently proposed and discussed XGBoost-based one, are able to capture complex interactions that arise due to structural flexibility, chemical complexity, and intermolecular forces such as hydrogen bonding. The model can incorporate these complexities without explicit physical modeling. As has been shown here the model is able to keep low prediction errors across different solvents, showing that the model is able to generalize between chemically diverse systems.

On the other hand, these models rely on a judicious selection of physical descriptors. Evaluating possible physical descriptors as input parameters is important to understand the relations between the properties and the solubility behavior. In this study, only two solvent descriptors (dielectric constant and boiling point) were used. It is suggested that future work investigates a broader base of properties, in particular solvent-specific properties, in order to enhance prediction accuracy and develop deeper understanding of the roles of possible parameters.

It is worth mentioning the importance of the effect of how the temperature-dependence of solubility is treated on model reliability. In this study it was observed that the temperature-dependence of the solubility was robustly and accurately captured by the XGBoost-based model, due to the incorporation of a monotonic-increase constraint into the model. This is crucial for process design in the pharmaceutical industry, where accurate temperature-dependence of solubility is critical.

As regards the chemical systems evaluated, it is noteworthy that one compound in particular, risperidone, exhibits very low absolute values of solubility in the evaluated solvents. This wide range in mole fraction solubility between different systems highlights the still little-understood role of certain molecular complexity and interactions between API and solvent. In future works, further investigation and refinement of intermolecular descriptors should be undertaken, in order to improve the modelling capability and generalizability across chemically diverse systems. It is worth mentioning that, in order to accurately train models for solubility data exhibiting wide solubility ranges, using logarithmic values is recommended.

Overall, the application of the XGBoost framework for solubility prediction of pharmaceutical compounds indicates a promising, potentially game-changing direction for computational drug formulation. Machine leaning methods such as XGBoost should be able to overcome limitations of conventional thermodynamic models, in order to be used as a more accurate tools for solvent selection and phase diagram modelling.

## Conclusions

In this work the mole fraction solubility of five organic solute molecules in various organic solvents at different temperatures has been calculated and predicted using a machine learning model based on the XGBoost framework. The overall RMSLE for solubility modelling of the four solutes of the training + test dataset across different solvents is 0.08. In the case of pure prediction of solubility for a compound outside the training set, butamben, the resulting RMSLE obtained is 0.41, with RMSLEs in methanol, 1-propanol, 2-propanol, 1-butanol and toluene of 0.22, 0.25, 0.31, 0.60 and 0.65, respectively. In comparison with semi-predictive models based on Flory-Huggins and NRTL-SAC theory, the XGBoost-based model consistently outperforms the other models for the same compounds, with less variability of error across solvents for each solute. The results show that machine learning models have a definite edge over semi-predictive models for modelling and prediction of solubility of pharmaceutical API-type molecules in organic solvents.

## Electronic Supplementary Material

Below is the link to the electronic supplementary material.

Supplementary Material 1

## Data Availability

All data generated or analysed during this study are included in this published article or in its supplementary information file.
